# Beverage industry TV advertising shifts after a stepwise mandatory food marketing restriction: achievements and challenges with regulating the food marketing environment

**DOI:** 10.1017/S1368980023002872

**Published:** 2023-12-27

**Authors:** Fernanda Mediano Stoltze, Teresa Correa, Camila Luz Corvalán Aguilar, Lindsey Smith Taillie, Marcela Reyes, Francesca Renee Dillman Carpentier

**Affiliations:** 1 Carolina Population Center, University of North Carolina, Chapel Hill, NC, USA; 2 School of Psychology, Pontificia Universidad Católica de Chile, Santiago, Chile; 3 School of Communication, Diego Portales University, Santiago, Chile; 4 Institute of Nutrition and Food Technology, University of Chile, Santiago, Chile; 5 Department of Nutrition, Gillings School of Global Public Health, University of North Carolina, Chapel Hill, NC, USA; 6 Hussman School of Journalism and Media, University of North Carolina, Chapel Hill, NC 27599, USA

**Keywords:** Child-directed marketing, Food marketing regulation, Food environment, Beverage industry

## Abstract

**Objective:**

Sugar-sweetened beverages (SSB) are heavily advertised globally, and SSB consumption is linked to increased health risk. To reduce unhealthy food marketing, Chile implemented a regulation for products classified as high in energies, sugar, saturated fat or sodium, starting with a 2016 ban on child-targeted advertising of these products and adding a 06.00–22.00 daytime advertising ban in 2019. This study assesses changes in television advertising prevalence of ready-to-drink beverages, including and beyond SSB, to analyse how the beverage industry shifted its marketing strategies across Chile’s implementation phases.

**Design:**

Beverage advertisements were recorded during two randomly constructed weeks in April-May of 2016 (pre-implementation) through 2019 (daytime ban). Ad products were classified as ‘high-in’ or ‘non-high-in’ according to regulation nutrient thresholds. Ads were analysed for their programme placement and marketing content.

**Setting:**

Chile.

**Results:**

From pre-regulation to daytime ban, child-targeted, daytime and total ads decreased by 51·8 percentage points (p.p.), 51·5 p.p. and 61·8 p.p. for high-in products and increased by 62·9 p.p., 54·9 p.p. and 61·8 p.p. for non-high-in products (*Ps* < 0·001). Additionally, total ready-to-drink beverage ads increased by 5·4 p.p. and brand-only ads (no product shown) by 7 p.p.

**Conclusions:**

After the regulation implementation, ‘high-in’ ads fell significantly, but ‘non-high-in’ ads rose and continued using strategies targeting children and being aired during daytime. Given research showing that advertising one product can increase preferences for a different product from that same brand and product categories, broader food marketing regulation approaches may be needed to protect children from the harmful effects of food marketing.

Sugar-sweetened beverages (SSB henceforth) are one of the most relevant contributors of the daily energy intake among children and adults^(1)^. Children in Latin America have among the highest SSB consumption rates^(1)^, making SSB a major contributor to their weight gain and diabetes risk^(2)^. Despite detrimental health effects linked to SSB consumption, these beverages remain the most advertised food product globally^(3)^, and this advertising is shown to influence children’s food and beverage preferences^(4,5)^, consumption and BMI^(6)^.

Responding to this concern, the WHO and UNICEF are urging countries to reduce the marketing of food and beverages (foods henceforth) that contribute to children’s unhealthy diets through comprehensive and mandatory policies^(7,8)^. Chile has been leading efforts^(9,10)^ to regulate unhealthy food marketing with a phased restriction for products classified as above legally defined thresholds in energies, sugar, saturated fat or sodium (‘high-in’ products henceforth)^(11)^. See Table 1 for a regulation description^(12,13)^. Chile’s marketing ban is part of a comprehensive set of regulations, including an SSB tax, front-of-package warning labels and school sales restrictions for high-in products^(14)^. SSB reduction is of particular interest in Chile due to the high advertising presence of sodas^(15)^ and high consumption of sugary beverages among Chileans^(12,16)^, where one out of ten children consume SSB daily^(13)^.

**Table 1:** Marketing restrictions and nutrient thresholds of the Chilean food marketing and labelling regulation

Marketing restrictions for high-in food and beverages			
Restrictions		Phase 1 child-targeted restrictions	Phase 2 daytime restrictions
High-in products cannot be promoted using child-directed appeals in any media such as using child actors, cartoon characters including equity characters, children’s music, toys, references to children’s daily life, fantastic arguments about the product, toys, games, and ‘hooks’ unrelated to the product		✓	✓
High-in products cannot be advertised on television to attract children (television programmes made for children or attracting audiences in which > 20 % were children)		✓	✓
High-in products cannot be promoted or advertised during daytime (Advertise across all television from 06.00 to 22.00)			✓

Progressive thresholds for defining ‘high-in’ beverages in the Chilean law of food labelling and advertising.			
Nutrients of concern per 100 ml	Phase 1	Phase 2	Phase 3
Energy	≥ 100 kcal	≥ 80 kcal	≥ 87 kcal
Sodium	≥ 100 mg	≥ 100 mg	≥ 100 mg
Total sugar	≥ 6 g	≥ 5 g	≥ 5 g
Saturated fats	≥ 3 g	≥ 3 g	≥ 3 g

Phase 1 thresholds, implemented on June 26, 2016, were in effect for ads sampled in April–May 2017 and 2018. Phase 2 thresholds, implemented on June 26, 2018, were in effect for ads sampled in April–May 2019. Phase 3 of the regulation was implemented on June 26, 2019 and is not assessed in this study (ads under this effect would have been collected in April–May 2020).

Chile introduced its marketing restrictions in two phases, starting first in June 2016 with a ban from using child-directed appeals (e.g. characters, toys) to market high-in products and a ban from advertising high-in products in television programmes made for children or with a child audience composition of 20 % or more. Two years later (May 2018), Chile added a ban on all high-in product television advertisements (ads henceforth) from 06.00 to 22.00^(17)^. Research shows that the number of high-in ads across television and children’s exposure to these ads was significantly lower after phase 1 and even lower after 2018’s phase 2, compared to pre-regulation^(18–20)^. Sugary soda ads, the most frequently advertised high-in product category at pre-regulation, were among the most dramatic drops^(18)^. However, the total amount of food advertising on television did not dramatically change across implementation, which suggests advertising shifted to existing, reformulated or new products that did not meet ‘high-in’ criteria.

Chilean children have a high consumption of both sugary and sugar-free ready-to-drink (RTD) beverages that contain non-nutritive sweeteners^(21)^. The WHO has recently warned against using no-sugar sweeteners to replace free sugar because they are not associated with health benefits in children^(22,23)^. Furthermore, recent evidence suggests that high consumption of ultra-processed foods, which includes sugar- and artificially-sweetened beverages, might increase all-cause mortality^(24)^. Research also shows that food marketing effects can go beyond the advertised product to impact preferences for brands^(25)^ and food categories^(25)^, increasing children’s overall dietary intake^(26)^. For instance, advertising exposure to sugar-sweetened sodas has been found to increase demand for the advertised soda and also sugar-free sodas from the same brand^(27)^. Thus, the WHO (2023) has urged countries to incorporate no-sugar sweeteners into regulatory measures aimed at promoting healthy diets.

The Chilean regulation does not restrict the marketing of products containing no-sugar sweeteners. Therefore, Chile’s regulation provides an important opportunity for informing countries considering food marketing restrictions^(7)^, on how sugary and non-sugar-sweetened beverages ads change in a regulated context. In this study, we assess changes in RTD beverages before and after the two phases of the Chilean food marketing regulation. We focus on the entire RTD beverage category, which includes sodas, industrialised flavoured waters, fruit drinks, sport and energy drinks, 100 % juices and plain waters, to capture advertising shifts between the most highly consumed beverages in Chile^(12,13,16,21)^ and other products (e.g. plain waters, 100 % juices) in beverage companies’ portfolios. Prior research shows that beverage companies in Chile adapted to the regulation through product reformulation^(28)^. Assessing the features and frequency of all RTD beverage advertising allows us to examine whether reformulation was accompanied by new strategies in product promotions in the beverage sector^(29)^. We focus on television advertising because television has been the main advertising medium in Chile^(30)^, and Chilean children and adolescents still report television as the main medium where they are exposed to ads for unhealthy foods or drinks^(31)^.

The first aim of this study is to assess the prevalence of RTD beverage advertising for products qualifying as ‘high-in’ *v*. ‘non-high-in’ at pre-regulation (2016), regulation phase 1 (2017 and 2018) and regulation phase 2 (2019) based on marketing content and placement strategies used with the ads. We also examine changes in the overall prevalence of child-directed marketing strategies and promotional foci (e.g. sodas, fruit drinks, water) in RTD beverage advertising across the four years under study. To address these aims, we conducted a quantitative content analysis of beverage advertising and linked the advertised products with their beverage subcategory, brands, nutrient content, ingredients and regulation status. The second aim of this study is to examine the specific trajectories of two beverage brands across the four years to more fully understand shifts that occurred within the same brand. This examination includes an assessment of promoted products based on their sugar content to assess how shifts might have been informed by regulatory sugar thresholds.

## Methods

### Sample

In April-May of each year from 2016 to 2019, television programming was recorded from 06.00 to 12:00 on Chile’s four major broadcast networks (Mega, Chilevision, TVN, and Canal 13) and the four most popular cable networks among Chilean youth (Discovery Kids, Disney, Cartoon Network, and Fox), according to Kantar IBOPE Media. Television programming was obtained from Chile’s National Television Council. A stratified two-week random sample was built by drawing one randomly selected Sunday, one randomly selected Monday, one Tuesday, etc., from April and one from May. This sampling framework is widely used in content analysis as it has been shown to be more efficient than pure random or consecutive day sampling, as the stratified sample accounts for content variation within the typical week^(32)^. Advertising was analysed if it contained at least one RTD beverage, including sodas, industrialised flavoured waters, fruit/vegetable drinks, sports, energy drinks, teas, 100 % fruit/vegetable juice, plain, pure and mineral waters (water henceforth). We included advertisements for supermarkets, restaurants, delivery apps and beverage brands.

### Procedure

A team of Chilean-trained coders performed a quantitative content analysis of ads for food and non-alcoholic beverages placed during short breaks within and between programmes. Coders recorded each ad’s placement information (date, channel, and programme), identified the marketing strategies used in each ad (see Codes section below) and listed the products and brands featured in each ad (up to 4 per ad in 2016, up to 7 in 2017–2019). Intercoder reliability of marketing strategies was assessed each year in a subsample of ads, which showed acceptable levels of agreement (Cohen’s Kappa > 0·70). Then, each product was linked to its nutritional content based on a longitudinal Nutrition Facts Panel database for products available in Chilean supermarkets during the four years under study^(33)^. These Nutrition Facts Panel datasets included product names, brands, descriptions, ingredients, energies, sugar, sodium and saturated fat content per 100 grams. Nutrition Facts Panel datasets used in prior examinations of Chilean marketing^(19)^ were updated after refining product identification and matching procedures.

Based on the content analysis and Nutrition Facts Panel data linked to each advertised product, we selected two of the most advertised beverage brands to specifically examine their advertising trajectories. We provide a thorough description of this examination in the analysis section.

### Codes

A codebook was used to identify and record details about each ad’s marketing strategies, including details about the promoted products in each ad. Codes regarding promoted products are described below, followed by marketing placement and content.

#### Product-related codes

Ad relevance: Ads promoting any food or beverage product or brand were coded as ‘1’ for being a food/beverage ad and ‘0’ if no food or beverage was promoted. Food/beverage ads were then coded for whether they promoted any RTD beverage product or brand (=‘1’) or if they did not promote an RTD beverage (=‘0’).

Product focus: Ads promoting any specific RTD beverage product were coded as ‘1’ for product focus. Ads promoting a beverage brand without showing a specific product were coded as ‘0’ for brand focus (no product).

Company focus: RTD ads announced by a beverage company were coded as ‘1’ for being company-focused. Ads for supermarkets, restaurants or delivery services that simply featured one or more brands or products were coded as ‘0.’

Beverage brand: Coders typed the names of the brand (e.g. Coca-Cola) and sub-brand (i.e. the product name, such as Coca-Cola/‘Coke’ Original) of the promoted beverages in the ad, including any sugar-related claims in the name of the product (e.g. zero sugar, sugar-free, light).

Beverage category: Promoted RTD beverages were coded for their product category (soda, industrialised flavoured water, fruit/vegetable drink, sports or energy drink, tea, 100 % fruit/vegetable juice, water) using dummy variables for each category. For instance, an ad that featured at least one soda and no other beverages was coded as ‘1’ for the soda category and ‘0’ for the other categories. An ad that featured both soda and water was coded as ‘1’ for the soda category and ‘1’ for the water category and ‘0’ for the remaining categories.

‘High-in’ qualifications: According to the Chilean Regulation,^(34)^ a product was considered ‘high-in’ and therefore subject to regulation if it contained added sugar, sodium or saturated fats above government-defined thresholds per 100 ml^(17)^. Water and 100 % fruit and vegetable juices are exempt from the regulation. For each RTD ad, each promoted beverage was first assessed for its regulation status based on the nutrient content thresholds enforced during the ad’s data collection period, allowing us to assess compliance with the regulation (‘cross-sectional thresholds’, henceforth). Ads from the 2016 pre-regulation year were assessed based on the phase 1 thresholds to be implemented in the following year. Each ad was then coded as ‘1’ if it featured at least one high-in RTD beverage based on the cross-sectional threshold and coded as ‘0’ if no high-in product was specifically promoted in the ad. Next, beverages in each ad were assessed for regulation status based on the strictest nutrient content thresholds set for the final phase of the Chilean regulation (‘final thresholds’, henceforth). Each ad was then further coded as ‘1’ if it featured a high-in beverage based on the final thresholds and ‘0’ otherwise. See cut-off values in Table 1.

#### Marketing placement and content codes

Codes about marketing placement and content were developed based on the Chilean regulation’s definition of ‘child-directed’ marketing^(34)^ and coding protocols used in prior studies^(35)^. Marketing placement and content of each ad were recorded as follows.

Time placement: In phase 2 of the Chilean regulation, high-in ads were no longer allowed to be on television between 06.00 and 22.00. To evaluate the progress of the phase 2 implementation, all ads across the four years of data collection were coded as ‘1’ if shown between 06.00 and 22.00 (henceforth ‘daytime’) and ‘0’ otherwise.

Channel placement: Ads found in television channels broadcast for free over the air were coded as ‘1’ for over-the-air and ads in paid cable channels were coded as ‘0.’ Note that the sampled over-the-air channels served the general audience, including children, whereas the cable channels sampled (e.g. Discovery Kids) were primarily aimed at children.

Placement attracting child audiences: An ad was coded as ‘1’ for being in a programme attracting children if at least one of these two criteria were met: (i) the ad was aired in a programme listed as made for children by their producers, or (ii) 20 % of the programme audience were children 4–12 years old according to audience ratings provided by Kantar IBOPE Media. Otherwise, the ad was coded as ‘0.’

Child-directed appeals in the ad content: The Chilean regulation defines child-directed appeals as the use of children or child voices, licensed characters, animated animals and other characters, celebrities, athletes, promotional gifts, prizes, contests, interactive games, references to fantasy, school, play, popular child phrases and child life within the promotional content^(17)^. Ads that contained any of these appeals were coded as ‘1’ for having child-directed content. Ads were coded as ‘0’ if they featured none of these appeals.

Child-targeted designation: Finally, based on the Chilean regulation, any ad that either used one or more child-directed appeals or was placed in a programme attracting child audiences was coded as ‘1’ for being child-targeted and ‘0’ otherwise.

### Analysis

To evaluate the first aim, frequencies of high-in and non-high-in RTD beverage ads were described for each year of analysis (2016–2019) in total and by whether the ads were child-targeted overall, specifically used child-directed appeals in the ad, were specifically placed in programmes attracting children, or were shown during the daytime (06.00–22.00). High-in designations using the cross-sectional nutrient content thresholds enforced each year were used to evaluate compliance with the regulation as it was implemented at that time. High-in designations using final thresholds were used to assess the overall quality of RTD beverage advertising. We also examined, for each of the four years, the frequency of RTD ads overall based on marketing content and placement strategy, as well as based on product subcategory (e.g. soda, sports/energy drink, water) and product focus (specific product(s) shown rather than a brand ad with no products shown). We also documented the frequency of high-in and non-high-in RTD beverage ads shown on over-the-air *v*. cable channels (only the final threshold is used for this documentation), as advertising shifts might differ based on audiences served and programmes offered (general audience *v*. child audience for over-the-air and cable, respectively). Frequencies reported are the average number of ads in one week of Chilean television. Also reported are percentages of specific types of ads (e.g. high-in ads with child appeals) out of the total number of weekly RTD beverage ads in that year.

Percentages between pre-regulation (2016) and the first year of phase 1 (2017) are compared using chi-square tests with subsequent pairwise comparisons to look for statistically significant changes immediately following the first implementation of the regulation. Percentages between the second year of Phase 1 (2018) and Phase 2 (2019) are compared to assess changes following the added ban on daytime high-in advertising. Percentages at pre-regulation (2016) are also compared with Phase 2 (2019) to provide an overall assessment of the marketing restrictions. Differences are assessed at *P* < 0·05 and expressed in percentage points (p.p.). Pairwise comparisons are adjusted using a Bonferroni correction.

To address the second aim, we assessed the advertising frequency of two specific soda brands and their sub-brands throughout the years. To do this, we first identified the fourteen soda brands featured in our sample. Then, we counted the number of times each soda brand appeared in an ad over the years and ranked them by frequency. We selected the top three advertised brands for analyses. We identified the sub-brands advertised, claims used, sugar content, and ingredients across the years, allowing us to find new products added to the brands’ portfolio and product reformulation. Sugar content examinations consisted of the number of times products of different sugar levels (described in the Codes section) appeared at each regulation phase to identify how many times each brand advertised a product with sugar above, below, and just below regulation cut-off values. We confirmed our data with the soda company’s yearly reports, where new brands and product reformulations were announced. We finally present the results of the first and third most advertised soda brands, as these brands used two different strategies for adapting to the marketing regulation.

## Results

### Changes in high-in, non-high-in, and total ready-to-drink beverage ads

Table 2 shows the frequencies and percentages (out of total RTD beverage ads) of high-in and non-high-in ads in total and based on marketing content and placement strategy. Table 3 shows the total RTD beverage ads by marketing strategy, by product subcategory and by promotional focus (product rather than brand-only). As shown in Table 2, high-in ads decreased and non-high-in ads increased in total and based on marketing strategy after the Chilean regulation was implemented. When applying cross-sectional thresholds to qualify beverages as high-in, findings indicate high compliance with marketing regulation phases where percentages of high-in ads out of total RTD beverage ads dropped between pre-regulation and the first year of phase 1 (*Ps* < 0·001) and remained similarly low at phase 2. Using the final thresholds to qualify beverages, high-in beverage ads in total decreased by 38·1 p.p. in the first year of phase 1, 24·0 p.p. from the second year of phase 1 to phase 2 and 61·8 p.p. from pre-regulation to phase 2 (*Ps* < 0·001). These decreases were due to drops in both child-targeted and daytime high-in ads. Child-targeted ads decreased by 31·6 p.p. from pre-regulation to the first year of phase 1 when the child-targeted restrictions were in place. These ads fell by an additional 23·6 p.p. from the second year of phase 1 to phase 2 when the daytime restriction entered into force, for a total drop of 51·8 p.p. from pre-regulation to phase 2 (*Ps* < 0·001). High-in ads aired during the daytime decreased by 34·1 p.p. from pre-regulation to the first year of phase 1 and by 19·5 p.p. from the second year of phase 1 to phase 2, for a total 51·5 p.p. drop from pre-regulation to regulation phase 2 (*Ps* < 0·001). Commensurate increases in non-high-in ads in total, with child targeting, and during the daytime were found at each phase (*Ps* < 0·001). Changes in the prevalence of child-directed and daytime ads according to regulation status based on the final thresholds are also shown in Table 2.

**Table 2:** Frequencies and percentages of ready-to-drink (RTD) beverage advertisements found across one week of Chilean television by marketing strategies and regulation status

Advertisements			Phase 1 child-targeted regulation								
	Pre-regulation		Year 1		Year 2		Phase 2 dtime regulation		Phase 1.y1 *v*. Pre-reg	Phase 2 *v*. Pre-reg	Phase 2 *v*. Phase 1. y2
	*n*	%	*n*	%	*n*	%	*n*	%	%	%	%
Total RTD beverage ads	605		664		777		662				
Ads promoting at least one high-in RTD beverage											
Cross-sectional nutrient content thresholds											
Total ads promoting at least one high-in RTD beverages	353	58·4 %	31	4·8 %	23	3·0 %	18	2·8 %	**−53·6 %**	**−55·5 %**	−0·2 %
Child-targeted overall	291	48·1 %	23	3·5 %	22	2·9 %	16	2·5 %	**−44·6 %**	**−45·5 %**	−0·4 %
Child-directed appeals in ad	287	47·4 %	23	3·5 %	22	2·9 %	16	2·5 %	**−43·9 %**	**−44·9 %**	−0·4 %
Placed in programmes attracting children	63	10·4 %	3	0·5 %	1	0·1 %	7	1·1 %	**−10·0 %**	**−9·3 %**	**1·1 %**
Daytime placement	293	48·4 %	10	1·5 %	7	0·9 %	14	2·3 %	**−46·9 %**	**−46·1 %**	**1·3 %**
Final nutrient content threshold											
High-in RTD beverages	384	63·5 %	166	25·4 %	193	25·7 %	11	1·7 %	**−38·1 %**	**−61·8 %**	**−24·0 %**
Child-targeted overall	322	53·2 %	141	21·6 %	187	25·0 %	9	1·4 %	**−31·6 %**	**−51·8 %**	**−23·6 %**
Child-directed appeals in ad	318	52·5 %	139	21·3 %	187	25·0 %	9	1·4 %	**−31·2 %**	**−51·1 %**	**−23·6 %**
Placed in programmes attracting children	72	11·8 %	19	2·8 %	43	5·7 %	1	0·2 %	**−9·0 %**	**−11·7 %**	**−5·6 %**
Daytime placement	319	52·8 %	122	18·7 %	155	20·7 %	8	1·2 %	**−34·1 %**	**−51·5 %**	**−19·5 %**
Ads promoting at least one non-high-in RTD beverage											
Applied nutrient content thresholds in each period											
Non-high-in RTD beverages	252	41·7 %	633	95·2 %	754	97·0 %	644	97·2 %	**53·5 %**	**55·5 %**	0·2 %
Child-targeted overall	171	28·3 %	535	80·2 %	666	85·2 %	569	85·0 %	**51·9 %**	**56·7 %**	−0·3 %
Child-directed appeals in ad	166	27·4 %	520	77·9 %	629	80·2 %	565	84·3 %	**50·5 %**	**56·9 %**	**4·1 %**
Placed in programmes attracting children	55	9·0 %	165	23·3 %	283	34·1 %	195	24·2 %	**14·3 %**	**15·2 %**	**−9·9 %**
Daytime placement	215	35·6 %	560	84·0 %	656	83·8 %	524	85·1 %	**48·4 %**	**49·5 %**	1·2 %
Final nutrient content threshold											
Non-high-in RTD beverages	221	36·6 %	498	74·6 %	584	74·3 %	651	98·3 %	**38·0 %**	**61·7 %**	**24·0 %**
Child-targeted overall	140	23·2 %	417	62·1 %	501	63·2 %	576	86·1 %	**38·9 %**	**62·9 %**	**22·9 %**
Child-directed appeals in ad	135	22·3 %	404	60·1 %	464	58·2 %	572	85·5 %	**37·8 %**	**63·2 %**	**27·3 %**
Placed in programmes attracting children	46	7·6 %	149	20·9 %	241	28·4 %	201	25·2 %	13·3 %	17·6 %	−3·3 %
Daytime placement	189	31·2 %	448	66·9 %	508	64·1 %	576	86·1 %	**35·7 %**	**54·9 %**	**22·0 %**

Phase 1.y1 is Year 1 and Phase 1.y2 is Year 2 of the first phase of regulation implementation consisting of child-targeted restrictions. Regulation status was calculated based on the nutrient content threshold in force in each period for the values under the title Cross-sectional nutrient content thresholds and based on the final thresholds for the values under the title Final nutrient content thresholds. Percentage of ads was calculated based on the total of ads featuring at least one RTD beverage. Differences were calculated with chi-square tests, *P* < 0·05. Pairwise comparisons were adjusted using the Bonferroni correction. Values in bold indicate statistically significant differences.

**Table 3:** Frequencies and percentages of ready-to-drink (RTD) beverage advertisements found across one week of Chilean television by marketing strategies and subcategories

Advertisements			Phase 1 child-targeted regulation								
	Pre-regulation		Year 1		Year 2		Phase 2 dtime regulation		Phase 1.y1 *v*. Pre-reg	Phase 2 *v*. Pre-reg	Phase 2 *v*. Phase 1. y2
	*n*	%	*n*	%	*n*	%	*n*	%	Difference (p.p)	Difference (p.p)	Difference (p.p)
Food and beverage ads	2892		2805		3144		2515				
Any RTD beverage-related ad	605	20·90 %	664	23·70 %	777	24·70 %	662	26·30 %	**2·8 %**	**5·4 %**	1·6 %
Child-targeted overall	462	76·3 %	558	84·0 %	688	88·6 %	585	88·4 %	**7·7 %**	**12·1 %**	−0·2 %
Child-directed appeals in ad	452	74·7 %	543	81·8 %	651	83·8 %	581	87·8 %	**7·1 %**	**13·0 %**	4·0 %
Placed in programmes attracting children	118	19·4 %	168	25·2 %	284	36·5 %	191	29·3 %	**5·8 %**	**9·9 %**	**−7·2 %**
Daytime placement	508	83·9 %	570	85·8 %	662	85·3 %	584	88·2 %	**1·9 %**	**4·3 %**	2·9 %
RTD beverage product *v*. brand	605	100 %	651	98·0 %	749	96·5 %	616	93·1 %	**−2·0 %**	**−6·9 %**	**−3·4 %**
Company *v*. others advertiser	462	76·40 %	495	74·50 %	607	78·20 %	498	75·20 %	−1·9 %	−1·2 %	−3·0 %
Soda	462	76·3 %	437	65·8 %	597	76·8 %	561	84·7 %	**−10·5 %**	**8·5 %**	**7·9 %**
Fruit/vegetable drink	26	4·2 %	139	20·9 %	98	12·6 %	68	10·2 %	**16·7 %**	**6·0 %**	−2·4 %
Sport and energy drinks	48	7·9 %	21	3·1 %	46	5·9 %	32	4·8 %	**−4·8 %**	**−3·1 %**	−1·0 %
Industrialised flavoured water	0	0·0 %	52	7·8 %	58	7·4 %	0	0·0 %	**7·8 %**	0·0 %	**−7·4 %**
100 % fruit/vegetable juice	0	0·0 %	11	1·7 %	8	1·0 %	0	0·0 %	**1·7 %**	0·0 %	**−1·0 %**
Water	70	11·6 %	20	3·0 %	50	6·4 %	2	0·2 %	**−8·6 %**	**−11·3 %**	**−6·2 %**

Phase 1.y1 is Year 1 and Phase 1.y2 is Year 2 of the first phase of regulation implementation consisting of child-targeted restrictions. p.p. is percentage points. Percentage of ads was calculated based on the total of ads featuring at least one RTD beverage. Only the percentage of RTD beverage ads in total was calculated based on ads matched with nutritional data. Differences are expressed in percentage points and were calculated with chi-square tests, *P* < 0·05. Pairwise comparisons were adjusted using the Bonferroni correction. Values in bold indicate statistically significant differences.

**Fig. 1:**
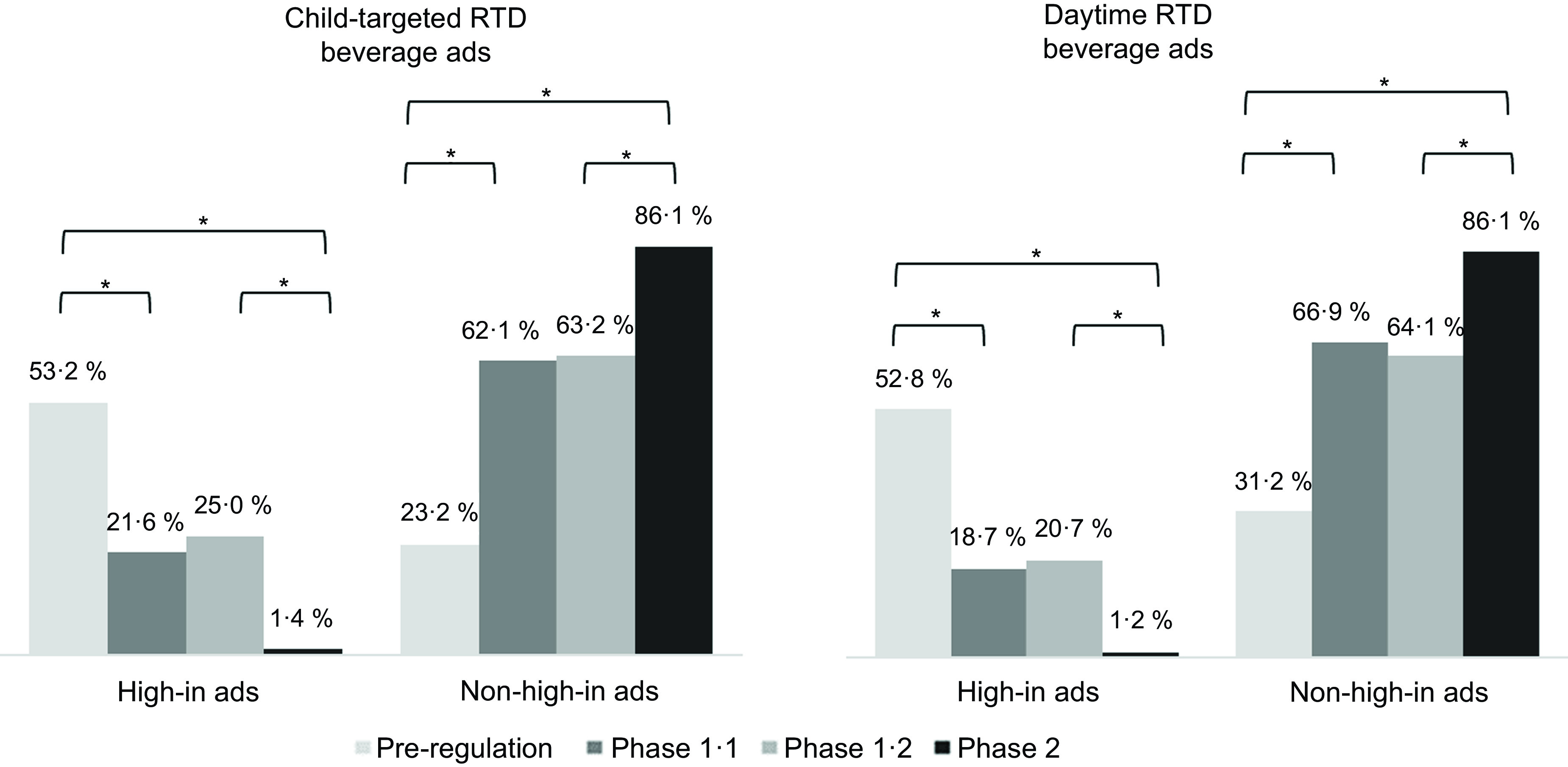
Prevalence of high-in and non-high-in ready-to-drink advertisements targeting children and aired during daytime hours across the two phases of the Chilean marketing regulation. Regulation status was calculated based on the nutrient content threshold in force in each period. Differences between (i) Phase 1·1 (Phase 1 Year 1) *v*. Pre-regulation, (ii) Phase 2 *v*. Phase 1·2 (Phase 1 Year 2) and (iii) Phase 2 *v*. Pre-regulation were tested with chi-square tests and post hoc pairwise comparisons adjusted using the Bonferroni correction. *P < 0·05

Table 3 shows RTD beverage ads overall (regardless of regulation status) represented 20·9 %, 23·7 %, 24·7 % and 26·3 % of all food ads aired per week at pre-regulation, first and second years of phase 1, and phase 2, respectively, with significant increases in these ads from pre-regulation to phases 1 and 2 (*Ps* < 0·001). This increase was primarily driven by child-targeted RTD beverage ads, which increased by 7·7p.p from pre-regulation to the first year of phase 1 (*P* < 0·001) and remained steady across post-regulation years for a total increase of 12·1 p.p from pre-regulation to phase 2 (*P* < 0·001). The percentage of RTD beverage ads aired during daytime hours remained similar across the years (*P* = 0·102). Analysed together, Tables 1 and 2 indicate a shift from high-in advertising as intended by the regulation, with important decreases after the daytime ban was implemented, to an increase in non-high-in advertising that included increases in child-targeted advertising. These shifts are illustrated in Fig. 1.

Table 3 further shows that increases in RTD beverage ads across regulation phases were driven by a rise in soda and fruit drink ads, which, compared to pre-regulation, increased by 8·5 p.p. and 6·0 p.p. in phase 2, respectively (*Ps* < 0·001). The percentage of ads featuring at least one soda represented 76·3 % of RTD beverage ads at pre-regulation and reached 84·7 % in phase 2. Plain bottled water and 100 % fruit/vegetable juice, the two product categories exempt from the regulation, were the least advertised across the years.

Another increase shown in Table 3 was in brand ads. That is, ads featuring specific products decreased by 2·0 p.p. from pre-regulation to the first year of phase 1, by additional 3·4 p.p. from the second year of phase 1 to phase 2 and by 6·9 p.p from pre-regulation to phase 2, which indicates brand ads increased 6·9 p.p. overall. We did not find significant differences in percentages of RTD beverage ads announced by a beverage company *v*. a supermarket, restaurant or delivery service (*P* = 0·107).

Finally, Table 4 explores specific differences in high-in and non-high-in advertising shifts in over-the-air *v*. cable television. Table 3 shows that the percentage of RTD beverage ads on over-the-air and cable television was similar at pre-regulation and regulation phase 2 (*P* = 0·840). Although the magnitude of changes from year to year varied between over-the-air and cable, both types of channels showed decreases in high-in ads and increases in non-high-in ads from pre-regulation to phase 2.

**Table 4:** Frequencies and percentages of ready-to-drink (RTD) beverage advertisements found across one week of Chilean television on over-the-air and cable television

Advertisements			Phase 1. Child-targeted regulation								
	Pre-regulation		Year 1		Year 2		Phase 2. Daytime regulation		Phase 1.y1 *v*. Pre-reg	Phase 2 *v*. Pre-reg	Phase 2 *v*. Phase 1.y2
	*n*	%	*n*	%	*n*	%	*n*	%	Difference (p.p)	Difference (p.p)	Difference (p.p)
Any RTD beverage product	1210		1301		1498		1232				
Over-the-air TV	659	54·5 %	626	48 %	773	52 %	676	54·9 %	**−6·3 %**	−0·5 %	3·4 %
Cable TV	551	45·5 %	675	51·9 %	738	49·3 %	556	45·1 %	**6·3 %**	−0·4 %	−4·1 %
High-in RTD beverages	769	63·5 %	331	25·4 %	385	25·7 %	21	2 %	−38·1 %	−61·8 %	−24·0 %
Over-the-air TV	489	63·6 %	188	56·8 %	257	66·8 %	18	85·7 %	**−6·8 %**	**22·1 %**	19·0 %
Cable TV	279	36·3 %	143	43·2 %	128	33·2 %	3	14·3 %	**6·9 %**	**−22·0 %**	−19·0 %
Non-high-in RTD beverages	443	36·6 %	970	74·6 %	1113	74·3 %	1211	98·3 %	38 %	62 %	24 %
Over-the-air TV	170	38 %	438	45 %	516	46 %	658	54 %	**6·8 %**	**16·0 %**	**8·0 %**
Cable TV	272	61 %	532	55 %	610	55 %	553	46 %	**−6·6 %**	**−15·7 %**	**−9·1 %**

Phase 1.y1 is Year 1 and Phase 1.y2 is Year 2 of first phase of regulation implementation consisting of child-targeted restrictions. p.p.is percentage points. Regulation status was calculated based on the final nutrient content threshold. Differences are expressed in percentage points and were calculated with chi-square tests, *P* < 0·05. Tests were adjusted for all pairwise comparisons using the Bonferroni correction. Values in bold indicate statistically significant differences.

### Examination of two brands

We looked closely at two of the most advertised soda brands to describe how the increase in non-high-in ads of advertised beverages relates to the brands’ advertising strategies. Table 5 shows Brand A’s response focused exclusively on increasing advertisements of sugar-free products. At pre-regulation, Brand A advertised the original soda with 10·8 g/100 ml of sugar, zero and light sub-brands. These sugar-free sub-brands appeared in more than 50 % of Brand A ads. In phase1, Brand A advertised sugar-free sub-brands almost exclusively, with some non-compliance cases. Then, the company diversified its sugar-free options by introducing a ‘without sugar’ sub-brand, which was the most advertised in phase 2. Brand B’s strategy focused on advertising sugar-free and reduced-sugar sodas. At pre-regulation, Brand B’s regular and zero sub-brands appearances were even. Brand B advertised its zero sub-brand exclusively in the first year of regulation. In the second year, the regular soda, which had 12·1 g/100ml of sugar, was reformulated, introducing a ‘reduced in sugar’ version with 4·8g/100ml of sugar, just below the 5g regulation threshold implemented in phase 2. The reformulated product was advertised under Brand B’s regular version. The reformulation was communicated as a product characteristic but not as introducing a new sub-brand. In phase 2, most advertisements combined appearances of Brand B’s reformulated version and a new sugar-free sub-brand, ‘without sugar.’

**Table 5:** Brands and sub-brands number of appearances in TV ads per week. Several brands might have been promoted in one ad

Brands and sub-brands	Product description	Sugar grams	Ingredients	Brands and sub-brands number of appearances in TV ads per week. Ads might have advertised several brands.							
				Pre-reg		Phase 1·1		Phase 1·2		Phase 2	
				*n*	%	*n*	%	*n*	%	*n*	%
Brand A											
Any A				202		192		290		169	
A	With sugar	10·8	Carbonated water, sugar, caramel colour, phosphoric acid, natural flavours and caffeine.	119	59·1 %	10	5·2 %	7	2·4 %	0	0 %
A Light	Sugar-free	0	Carbonated water, caramel colour, phosphoric acid, natural flavours, Aspartame, Potassium benzoate, acesulfame potassium, caffeine and citric acid. Phenylketonurics: contains phenylalanine.	93	45·9 %	36	18·5 %	118	40·8 %	34	20·2 %
A Zero	Sugar-free	0	Carbonated water, caramel colour, phosphoric acid, aspartame, natural flavours, potassium benzoate, acesulfame potassium, sodium citrate, caffeine.	112	55·3 %	183	95·3 %	61	21·1 %	5	2·7 %
A Without sugar	Sugar-free	0	Carbonated water, caramel colour, phosphoric acid, aspartame, natural flavours, potassium benzoate, acesulfame potassium, sodium citrate, caffeine, Phenylketonurics: contains phenylalanine.	0	0 %	0	0 %	181	625 %	158	93·8 %
Brand B											
Any B				61		89		94		158	
B	With sugar	12·1	Carbonated water, sugar, citric acid, modified starch, potassium benzoate, natural and artificial flavour flavours, S.A.I.B., sunset yellow, red Allura AC, DSS and BHA.	33	53·7 %	0	0 %	0	0 %	0	0 %
B	Reduced sugar	4·6	Carbonated water, sugar, citric acid, natural and artificial flavour, sucralose, modified starch, potassium benzoate, acesulfame potassium, Sodium gluconate, S.A.I.B. sunset yellow, red Allura AC, DSS and BHA.	0	0 %	0	0 %	93	98·4 %	36	22·8 %
B Zero	Sugar-free	0	Carbonated water, citric acid, sodium citrate, potassium sorbate, aspartame, gum acacia, natural and artificial flavours, potassium benzoate, acesulfame potassium, SAIB, ascorbic acid (vitamin C), sunset yellow, EDTA, DSS and red Allura AC. Phenylketonurics: contains phenylalanine.	28	46·3 %	89	100 %	75	79·3 %	23	14·6 %
B Without sugar	Sugar-free	0	Carbonated water, citric acid, sodium citrate, potassium sorbate, Aspartame, gum acacia, natural and artificial flavours, potassium benzoate, acesulfame potassium, SAIB, ascorbic acid (vitamin C), sunset yellow, EDTA, DSS and red Allura AC. Fenilcetonuricos: contains phenylalanine.	0	0 %	0	0 %	0	0 %	133	84·2 %

Percentage of specific sub-brand appearances are over the total of the brand (Any A or Any B) appearances on television advertisements. Percentages sum up more than 100 % as several sub-brands might have been promoted in the same ad.

## Discussion

This study assessed changes in the television advertising of RTD beverages in Chile after the child-targeted and daytime food and beverage marketing regulations were implemented. Our findings indicate that the beverage industry significantly reduced its advertising of high-in beverages. Child-targeted ads, ads aired during the day and total ads promoting high-in RTD beverages (promoting high-in and non-high-in products) decreased significantly after each regulation phase, suggesting substantial improvements in the children’s food marketing environment. These results support the evidence on the feasibility^(10,36)^ and positive impact of statutory food marketing regulations^(8)^.

We also found that advertisements of RTD beverages in total and those targeting children increased significantly in phase 1 and phase 2 compared to pre-regulation, whereas the percentage of daytime RTD beverage ads remained similar over the years under study. The increase in RTD beverage ads was driven by the shift to advertising non-high-in RTD beverages, especially child-targeted and aired during the daytime, which increased significantly in each regulation phase. These results suggest that instead of promoting high-in beverages by targeting older audiences or placing ads after 22.00, companies shifted to promote almost exclusively non-high-in RTD beverages to continue targeting children.

This implied an increase in the total and child-targeted RTD beverage ads in phases 1 and 2 compared to pre-regulation. These results align with literature that suggest children and adolescents are a key segment for food companies.^(37,38)^, as they have purchase power and the ability to influence family spending (‘pester power’)^(37,39)^. Furthermore, given young vulnerability to marketing, companies can foster early relationships between children and brands, shaping the preferences of future adult consumers^(40)^. This might partially explain the fact that beverage companies adapted their marketing strategy in order to continue to appeal to the young.

### Changes in ads promoting high-in ready-to-drink beverages

In the first phase of the food marketing regulation, when child-targeted advertising restrictions were in place^(17)^, high-in ads targeting children and aired during the day decreased by a significant 31·6 % and 34·1 %, respectively. Additionally, high-in ads in total decreased by 38·1 %, despite the regulation not banning high-in ads in itself in phase 1. This suggests that child-targeted restrictions can reduce the use of child-targeted strategies and high-in beverage advertising in total. Our results also suggest that these changes were driven by the decrease in total high-in beverage ads rather than the decrease in the use of child-targeted strategies in ads promoting high-in beverages, as the regulation established.

Comparing the second year of phase 1 to phase 2, when the daytime restriction was added, child-targeted high-in ads decreased by an additional 23·6 %, and high-in daytime ads by an additional 19·5 %. Also, high-in ads in total decreased by an additional 24 %. These findings show that the combination of child-targeted and daytime restrictions had a more substantial effect on reducing high-in RTD beverage advertisements on television than child-targeted restrictions only, supporting the calls to restrict exposure and power of food marketing^(7)^.

### Changes in ads promoting ready-to-drink beverages in general and non-high-in ready-to-drink beverages

We found that using child-directed appeals, such as cartoons, was the main strategy used to target children in RTD beverage ads. This suggests companies include child-appealing strategies in RTD beverage ads placed in child and general audience programmes to capture children’s attention, given that children mostly consume general audience programming^(41)^. This aligns with studies showing children are the main target of non-core foods marketing^(42)^. We also identified that ads promoting beverage brands not linked to specific beverage products had a small but significant increase across the years. The Chilean regulation applies marketing restrictions based on product nutrient content^(36)^, and thus, brand marketing not linked to a product is exempted from regulation. The increasing tendency in brand-focused beverage advertising should be monitored as children and adolescents can be highly influenced by food brands^(6,25)^ and by the marketing spill-over effects^(25)^.

We found that despite the increase of RTD beverage ads across the years, the frequency of advertised beverage subcategories remained stable, with sodas representing the most advertised RTD beverage, fruit drinks increasing in advertising presence, and water and 100 % juice ads being rarely advertised across the years. Instead, the industry’s primary response to the marketing regulations was advertising the same product subcategories by shifting to sugar-free and reduced-sugar beverages. This pattern in beverage advertising aligns with research showing that, in a regulated context, consumers substitute from ‘high-in’ to ‘non-high-in’ products or products with fewer warnings within the same product category^(43,44)^. These results are also coherent with a study showing that after Chile implemented the set of food policies, high-in beverage purchases decreased by 23·7 %, whereas non-high-in beverage purchases increased by 4·8 %^(45)^.

### Advertising trajectories of two beverage brands

The analyses of two soda brand advertisements across the years showed different strategies to adapt to the marketing regulations. One of the analysed brands (Brand A) shifted to advertise existing and new sugar-free products while keeping the full sugar product on shelves. In contrast, the other brand (Brand B) reformulated its sugary beverage reducing sugar just below regulation cut-off values, discontinuing the full sugar version and promoting reduced-sugar and sugar-free products. Brand B’s reformulation was communicated as a new product characteristic rather than a new sub-brand, enabling the company to advertise Brand B’s reduced-sugar version using the same brand image as before the regulation was implemented.

This strategy to reduce sugar to just below regulation cut-off values in some products, such as the Brand B’s reduced in sugar version, was already reported in a different study^(28)^ that showed minor differences in overall energy or nutrient distributions from pre- to post-regulation across food categories. These findings resonate with studies that describe RTD beverage companies have diversified their portfolios with new and reformulated products to address public concerns about SSB and regulatory pressures^(46)^. More research is needed to understand the effect of these marketing strategies on children^(46)^ and their alignment with the regulations’ ultimate goals.

### Policy implications

The overall analysis of the beverage industry’s response to the marketing regulation shows that along with significant reductions in high-in beverage ads, the presence of RTD beverage ads in total increased, as well as the use of child-targeted strategies after the marketing regulations, was implemented. The products promoted were sugar-free and reduced in sugar, which implies a relevant improvement in the marketing food environment. However, International Nutritional Guidelines emphasise increasing children’s water consumption^(47)^, which is concerningly low globally^(48)^. Therefore, to ensure that food marketing regulations comply with the current guidelines of the WHO, countries should aim to reduce, at the very least, the exposure to sugary and sugar-free beverages and, at the same time, to implement effective policies to promote water consumption^(47)^.

The Chilean regulation is considered the most comprehensive food marketing policy implemented to date^(9)^. This and prior studies^(19)^ show the effectiveness of advertising restrictions in reducing child-targeted marketing of non-recommended foods on television. However, even in one of the best current regulated scenarios, this approach might be insufficient to protect children from food marketing^(10)^, not just because unhealthy food marketing migrates from regulated to non-regulated media^(49)^, but because marketing within certain non-banned food categories might also influence children’s unhealthy preferences^(50)^. From this perspective, restrictions should move forward to regulate product categories and brands more broadly, aiming to reduce total food marketing exposure to children.

Importantly, the compliance we found for RTD beverage ads in phase 1 was higher than for the food and beverages ads reported in one of our previous studies assessing the Chilean regulation^(15)^, which might be associated with the nutritional content variability and potential for reformulation of beverages^(46)^. Thus, these results only represent RTD beverage ads. Also, we identify child-targeted ads based on the Chilean regulation definition, which is limited as it does not consider some strategies likely child-appealing^(7)^. Given that we analysed two weeks of television advertising, we could have missed other advertised products or marketing strategies. We diminished that risk by using a randomly constructed weeks sampling to account for content variation^(32)^. Additionally, our findings only represent the shifts in RTD beverage television advertising and cannot be extrapolated to other media.

### Conclusion

After the stepwise Chilean food marketing regulation was fully implemented, advertisements promoting high-in RTD beverages were almost absent on television, showing a significant decrease in the sugar content of advertised beverages. When the child-targeted and daytime restrictions were combined, the prevalence of high-in RTD beverage advertisements targeting children and aired during the day reached its lowest level. This supports the evidence suggesting marketing restrictions are likely to be most effective when they are mandatory, linked to a defined nutrient profile, and include television advertising restrictions extending beyond children’s programming^(8)^, as established in Chile’s food marketing restriction.

However, we simultaneously observed that the RTD beverage industry shifted to promote non-high-in sugar-free and reduced-sugar beverages and showed a slight increase in the use of brand advertising across the years with greater child-targeted promotion. As the consumption of RTD beverages might have long-term detrimental effects^(22–24)^, and food marketing effects operate at brand and product category levels^(25,26)^, RTD beverage marketing in general could have detrimental effects on children’s beverage preferences. Therefore, monitoring the nature, extent and effect of the emerging food marketing environment in regulated contexts is critical to inform countries working on food marketing restrictions. Research on more comprehensive and upstream approaches that could complement the positive effects of child-targeted and daytime restrictions is required to reduce food marketing exposure broadly.
